# A METHOD FOR MEASURING INTRA-TISSUE SWELLING PRESSURE USING A NEEDLE MICRO-OSMOMETER

**DOI:** 10.22203/eCM.v040a09

**Published:** 2020-09-20

**Authors:** C.M. Krull, A.D. Lutton, J.W. Olesik, B.A. Walter

**Affiliations:** 1 Department of Biomedical Engineering, The Ohio State University, Columbus OH, USA; 2 Trace Element Research Laboratory, School of Earth Sciences, The Ohio State University, Columbus, OH, USA; 3 Spine Research Institute, The Ohio State University, Columbus, OH, USA

**Keywords:** Osmotic pressure, intervertebral disc, osmolality, extrafibrillar water, mechanotransduction, Gibbs-Donnan

## Abstract

The intervertebral disc’s ability to resist load and facilitate motion arises largely from osmotic swelling pressures that develop within the tissue. Changes in the disc’s osmotic environment, diurnally and with disease, have been suggested to regulate cellular activity, yet knowledge of *in vivo* osmotic environments is limited. Therefore, the first objective of this study was to demonstrate proof-of-concept for a method to measure intra-tissue swelling pressure and osmolality, modeling micro-osmometer fluid flux using Darcy’s law. The second objective was to compare flux-based measurements of the swelling pressure within nucleus pulposus (NP) tissue against ionic swelling pressures predicted by Gibbs-Donnan theory. Pressures (0.03–0.57 MPa) were applied to NP tissue (*n* = 25) using equilibrium dialysis, and intra-tissue swelling pressures were measured using flux. Ionic swelling pressures were determined from inductively coupled plasma optical emission spectrometry measurements of intra-tissue sodium using Gibbs-Donnan calculations of fixed charge density and intra-tissue chloride. Concordance of 0.93 was observed between applied pressures and flux-based measurements of swelling pressure. Equilibrium bounds for effective tissue osmolalities engendered by a simulated diurnal loading cycle (0.2–0.6 MPa) were 376 and 522 mOsm/kg H_2_O. Significant differences between flux and Gibbs-Donnan measures of swelling pressure indicated that total tissue water normalization and non-ionic contributions to swelling pressure were significant, which suggested that standard constitutive models may underestimate intra-tissue swelling pressure. Overall, this micro-osmometer technique may facilitate future validations for constitutive models and measurements of variation in the diurnal osmotic cycle, which may inform studies to identify diurnal- and disease-associated changes in mechanotransduction.

## Editor’s note

The Scientific Editor responsible for this paper was Sibylle Grad.

## Introduction

Between adjacent vertebrae within the spine sits the intervertebral disc (IVD), a structure which provides flexibility while transferring loads through the spinal column. The disc’s load-bearing capacity is governed by the hydration that is maintained under a given load within the individual tissue regions that comprise it. This hydration is determined by the proteoglycan (PG) content within each region. PGs impart tissue a negative fixed charge density (FCD) (Urban and Maroudas, 1979b); and consequently, their presence generates an uneven distribution of ions between the tissue and interstitial fluid, as described by Gibbs-Donnan equilibrium (Urban *et al*., 1979a; Lai *et al*., 1991; Gu *et al*., 1997; Gooch and Tennant, 1997). Together with the concentration of tissue matrix molecules, these intra-tissue ions engender an osmotic swelling potential that causes the tissue to imbibe water and ultimately determines the disc’s propensity to resist a given load (Urban and Maroudas, 1981; Vergroesen *et al*., 2018; Emanuel *et al*., 2018).

Physiologically, the IVD experiences load in a diurnal cycle, with lower loads occurring during prone sleep and higher loads occurring during daytime activity (Wilke *et al*., 1999; Sivan *et al*., 2006b). These changes in load compel changes in the disc’s hydration, such that higher loads drive water out of the tissue. Fluctuations in hydration in turn engender an osmotic environment within the disc that cycles with applied loads. During the progression of age and degeneration, the tissue loses PGs, thus shifting toward a more fibrous and less-hydrated structure. These degeneration-induced changes in composition have been suggested to significantly alter the magnitude and kinetics of the disc’s diurnal osmotic cycle (Johannessen and Elliott, 2005; Perie *et al*., 2006; Massey *et al*., 2012; Paul *et al*., 2018; Yang and O’Connell, 2019).

Such changes have widespread ramifications, as the osmotic environment influences both the disc’s mechanical behavior and the metabolism of its cells (Stokes and Iatridis, 2004; Haschtmann *et al*., 2006). Fluctuations in osmolality have been shown to induce cellular changes in volume (Haider *et al*., 2006; Wang *et al*., 2015; Zelenski *et al*., 2015), shape, chromatin condensation (Irianto *et al*., 2013), gene expression (Boyd *et al*., 2005; Wuertz *et al*., 2007), cytoskeletal organization (Chao *et al*. 2006), ion channel activation (O’Conor *et al*., 2014; Walter *et al*., 2016), and matrix production (Ishihara *et al*., 1997; O’Connell *et al*., 2014; Krouwels *et al*., 2018). These findings suggest that cellular activity is directly linked to the diurnal- and disease-associated changes in tissue osmolality. However, while many studies have demonstrated the importance of the osmotic environment within cartilaginous tissues such as the IVD and articular cartilage, the osmotic conditions studied *in vitro* do not yet sufficiently simulate the cellular environment *in vivo*. Thus far, many *in vitro* studies specifically investigating the biological response of cells to osmotic loading have applied static osmotic loads, and a few have investigated the effects of relatively sudden leaps in osmolality. Meanwhile, *in vivo*, the osmolality of the disc changes substantially throughout the day, although such changes occur slowly due to the low permeability of the tissue (Urban and McMullin, 1985; Vergroesen *et al*., 2016; Emanuel *et al*., 2018). Part of this gap between *in vitro* and *in vivo* conditions exists because the magnitude and kinetics of the osmotic cycle, which vary based on individual disc health, body mass index, and activity levels (Urban and McMullin, 1985), remain poorly characterized. Thus far, measurements have occurred largely at equilibrium and under a limited range of applied loads. Ultimately, in order to understand how osmotic mechanotransduction is involved in the initiation and progression of disease, more comprehensive measures of the osmotic conditions disc cells experience under representative *in vivo* conditions (*i.e*., patient-specific magnitudes and dynamic changes) are necessary.

Therefore, the primary aim of this study was to evaluate a minimally invasive method for its potential to measure intra-tissue swelling pressures and osmolalities *in situ*. Importantly, development of such a method could be used to assess the osmotic environment that develops within the disc under simulated *in vivo* conditions. To this end, a micro-osmometer technique was adapted from a study by Sivan *et al*. (2013), which demonstrated that the FCD of NP tissue could be derived from a linear function of fluid flux from a micro-osmometer probe (greater flux into the tissue corresponded to greater FCD). Using this technique, the aim was to determine whether modeling the fluid flux using Darcy’s law could provide a measurement of intra-tissue swelling pressure. As an initial validation for this micro-osmometer technique, pressure was applied to isolated bovine NP tissue by equilibrium dialysis. While the annulus fibrosus and cartilage endplate impart boundary conditions for the osmotic environment of the NP, and their osmotic environments are of clear interest themselves, isolated bovine NP tissue was chosen for simplicity in order to demonstrate proof-of-concept for the method. The intra-tissue swelling pressures, determined from flux measurements, were then compared against the pressures applied during equilibration. A corollary aim of this study was to evaluate Gibbs-Donnan equations directly for their capability to approximate osmotic swelling within NP tissue. With this aim, the ionic component of osmotic pressure was determined using inductively coupled plasma optical emission spectrometry (ICP-OES) to measure intra-tissue sodium content, and Gibbs-Donnan theory to calculate FCD and intra-tissue chloride content. Ionic swelling pressures were then compared to the swelling pressures measured using micro-osmometer flux.

## Materials and Methods

### System described by Darcy’s equation

Tissue swelling pressures were measured by modeling fluid flux across the membrane of a microdialysis probe using Darcy’s law, which describes the pressure-driven flux of a fluid (*q*) through a porous medium (Darcy, 1856),

$$q=\frac{-k_{D}A}{L_{D}\mu }(\Delta P).$$

In this equation, variables are represented as follows: permeability of the medium by *k*_*D*_, cross-sectional area of flow by *A*, length of the medium by *L*_*D*_, viscosity of the permeating fluid by *μ*, and net pressure gradient by *ΔP*.

**Fig. 1.**
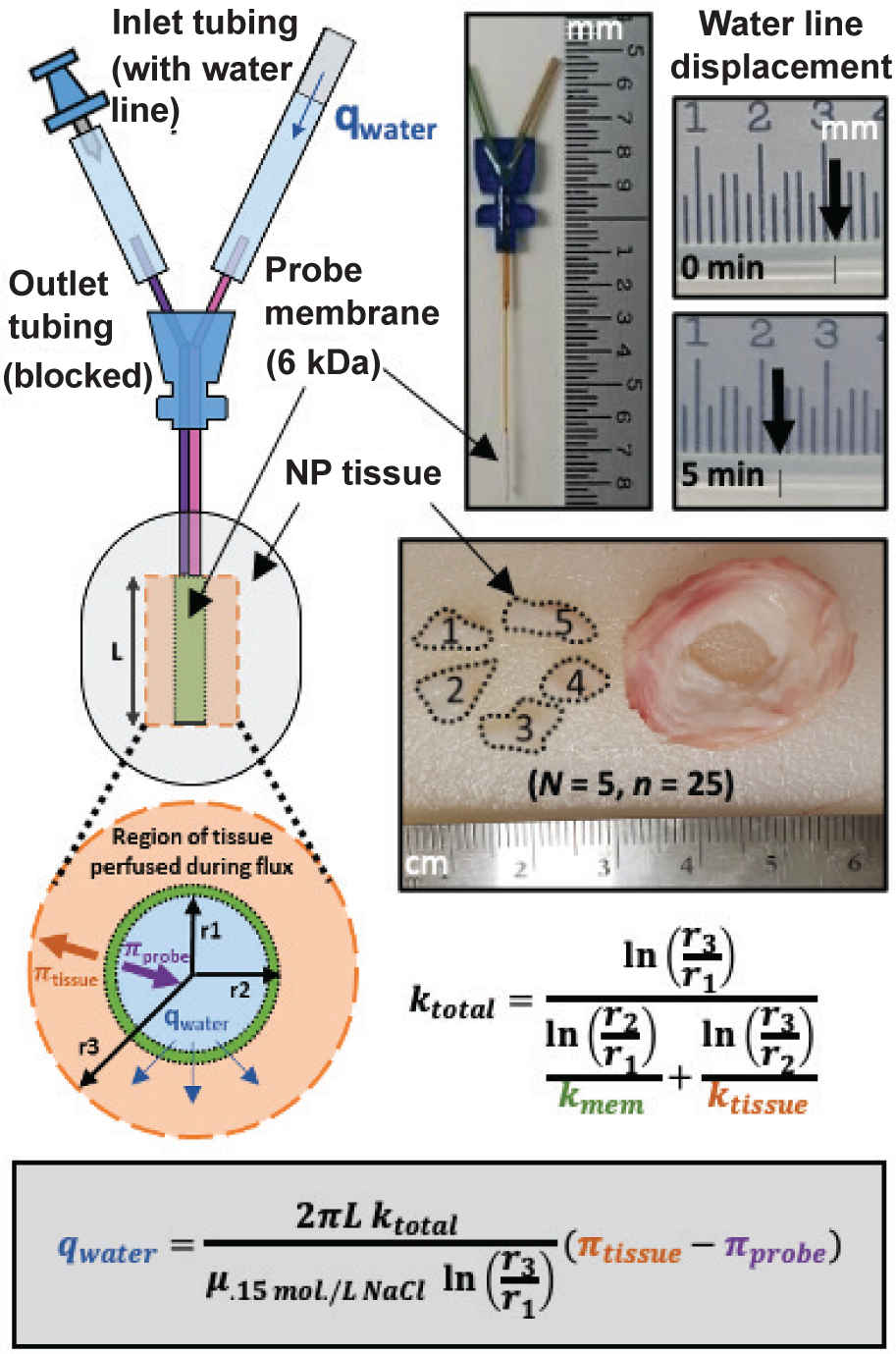
System schematic. Depiction of probe-tissue microdialysis, with variables defined for application of Darcy’s law for radial flow.

The suggested system, (shown in Fig. 1), consisted of a cylindrical membrane inserted into tissue, and could be described by the radial form of Darcy’s law,

$$q_{\text{water}}=\frac{2\pi L\;k_{\text{total}}}{\mu_{.15\;\text{mol}/L\;\text{Nacl}}\;\text{ln}\;\left(\frac{r_{3}}{r_{1}}\right)}\left(\pi_{\text{tissue}}-\pi_{\text{probe}}+P_{\text{tissue}}-P_{\text{air}}\right)$$

where the total pressure gradient (*ΔP*) driving flow is specified as the sum of osmotic and hydrostatic pressure gradients between the tissue and probe. For these experiments, the osmotic pressure differential was defined as the difference between the osmotic pressure of the tissue adjacent to the probe (*π*_*tissue*_) and the osmotic pressure of the 0.15 mol/L NaCl solution filling the probe (*π*_*probe*_). Meanwhile, the hydrostatic pressure gradient was defined as the difference between the hydrostatic pressure within the tissue and that within the tubing, which was open to the air. For these experiments, across the range of tissue densities, the hydrostatic pressure gradient (*P*_*tissue*_ − *P*_*air*_) was measured to be 3 orders of magnitude lower than the osmotic pressure gradient, and was therefore assumed to negligibly affect flux. This result was consistent with previous studies, which demonstrate that the hydrostatic pressure gradient approaches zero at equilibrium (Park *et al*., 2003). With this simplification, the following equation was used to determine tissue swelling pressures:

$$q_{\text{water}}=\frac{2\pi L\;k_{\text{total}}}{\mu_{.15\;\text{mol}/L\text{ NaCl }}\text{ln}\;\left(\frac{r_{3}}{r_{1}}\right)}\left(\pi_{\text{tissue}}-\pi_{\text{probe}}\right).$$

To take flux measurements, a microdialysis probe was filled with 0.15 mol/L NaCl, which is the same salt concentration under which tissue was equilibrated. Therefore, the electric potential difference between probe and tissue, as well as the resulting net flux of ions, were considered negligible (Gu *et al*., 1997). The remaining chemical potential of the intra-tissue ions and matrix proteins was expected to drive the flux of water (*q*_*water*_) into the tissue. However, consistent with the theoretical consideration that even though only water is flowing, that water must flow through NaCl solutions on either side of the membrane. The viscosity of the permeating fluid (*μ*_*.15 mol/L NaCl*_) was defined to be that of 0.15 mol/L NaCl at room temperature (298.15 K) (Zhang and Han, 1996). In these equations, *L*, r_1_ and *r*_*2*_ represent the probe membrane’s length, inner radius, and outer radius, respectively. Meanwhile, *r*_*3*_ represents the theoretical radius of perfusion - the cylindrical region of tissue perfused during flux (Fig. 1). The effective permeability (*k*_*total*_) of the entire region perfused during flux, including both membrane and tissue, was calculated as:

$$k_{\text{total}}=\frac{\text{ln}\;\left(\frac{r_{3}}{r_{1}}\right)}{\frac{\text{ln}\;\left(\frac{r_{2}}{r_{1}}\right)}{k_{\text{mem}}}+\frac{\text{ln}\;\left(\frac{r_{3}}{r_{2}}\right)}{k_{\text{tissue}}}},$$

which is the harmonic average of the individual membrane (*k*_*mem*_) and tissue (*k*_*tissue*_) permeabilities (Ahmed, 2010). To make this calculation, membrane permeability was measured empirically, as described below. Meanwhile, tissue permeability, which is known to be influenced by the degree of tissue strain (Holmes, 1985; Punter *et al*., 2020), was approximated from an equation describing the relationship between the deformation and permeability of bovine NP tissue (Heneghan and Riche 2008a),

$$k_{\text{tissue}}(\lambda )=1.59\times 10^{-15}{\left(\frac{\lambda -0.2}{0.8}\right)}^{1.13}e^{\left[-\frac{0.02\left(\lambda^{2}-1\right)}{2}\right]}.$$

This equation defines tissue permeability in terms of the stretch ratio, *λ* = *h*_*f*_/*h*_*o*_ where *h*_*o*_ denotes the initial height of the specimen and *h*_*f*_ denotes the final height after confined compression. For the current study, pressure was applied isotropically to the tissue; therefore, it was assumed that tissue compression proceeded radially. For that reason, changes in tissue radius were considered a more appropriate measure of stretch, and *r*_*f*_/*r*_*o*_ was used in place of the one-dimensional stretch ratio. Tissue radii at post-excision (*r*_*o*_) and post-equilibration (*r*_*f*_) were calculated from wet weights at the respective time point assuming spherical geometry and tissue density of 1,000 kg / m^3^.

For all equations, outer membrane radius (*r*_*2*_) was given by the manufacturer (0.12 mm), membrane length (*L*) was measured using a precision ruler (5 mm), and inner membrane radius(*r*_*1*_) was measured using a microscope (0.113 mm). Radius of perfusion (*r*_*3*_) was approximated for each tissue individually by assuming that the cylindrical volume that the fluid occupied within the tissue was equivalent to the volume of fluid that perfused into the tissue (Fig. 1). This yields:

$$\pi L\left(r_{3}{}^{2}-r_{2}{}^{2}\right)=q_{\text{water}}t,$$

where *t* represents the total time of perfusion and was 5 min for all experiments. It was assumed that the timescale of the measurement (5 min) was much smaller than the time required for the perfused fluid to reach its internal equilibrium state. Therefore, for simplicity, the fluid was assumed to occupy space within the tissue equal to its volume.

### Probe membrane permeability

**Fig. 2.**
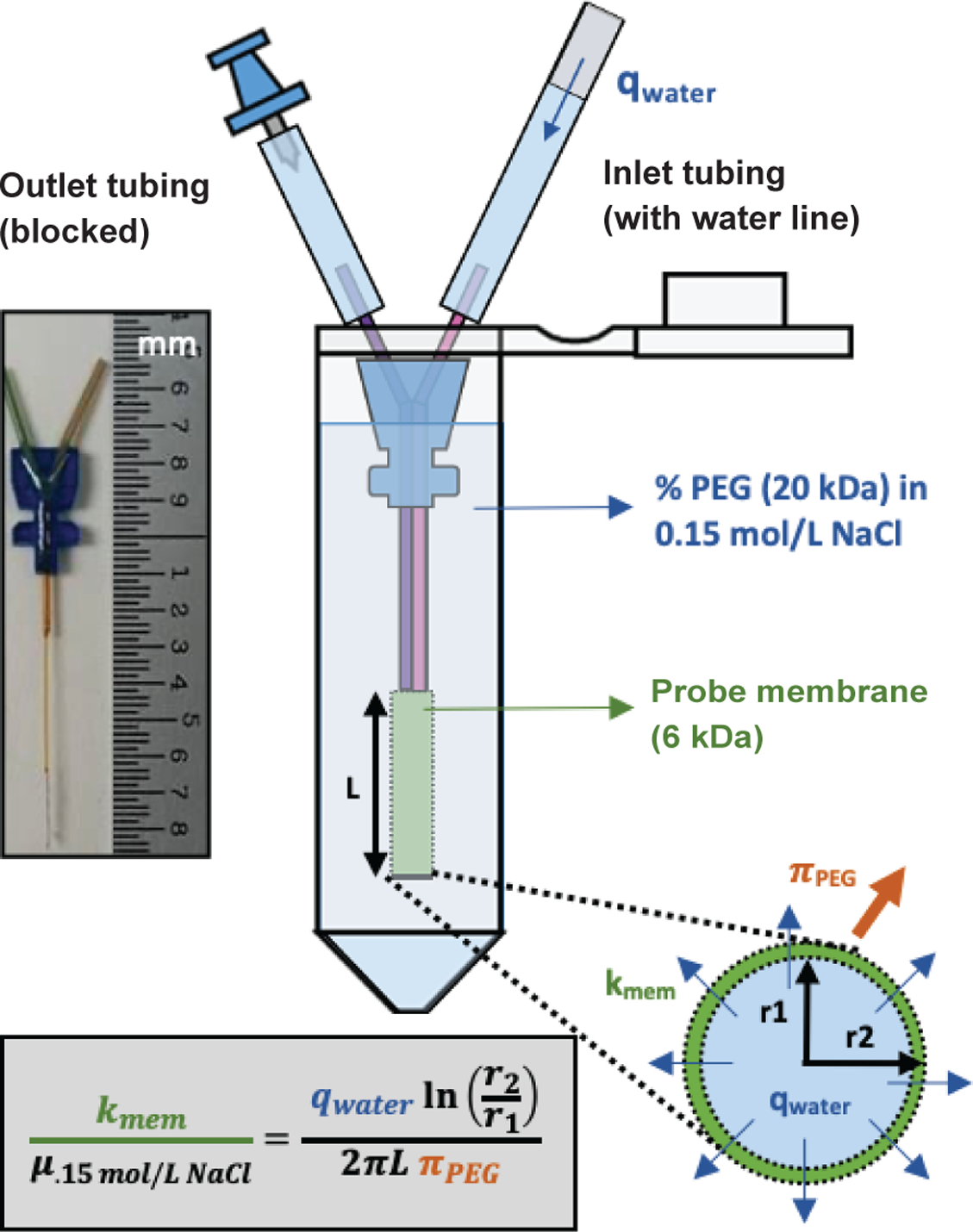
Probe membrane permeability. Schematic of setup for determining probe membrane permeabilities under known applied pressures, with variables defined for application of Darcy’s law for radial flow.

Membrane permeabilities were expected to change with pressure (Stevenson *et al*., 1978), and were therefore measured separately under known osmotic pressures. Micro-osmometer probes with 6 kDa cutoff polyethersulfone membranes (SciPro Inc. #MAB 4.15.4.PES, Sanborn, NY, USA), shown in Figs. 1 and 2, were hydrated in distilled water for 30 min, then tubing (Ismatec #EW-06460-14, Wertheim, Germany) was attached and flushed with 0.15 mol/L NaCl. Air was injected to create a visible water line, and the outlet tubing was blocked using a pushpin (Fig. 2). To approximate membrane permeability at each pressure applied to tissue, probes were placed in 5, 10, 15, 20 and 25 % (g/mL) 20 kDa polyethylene glycol (PEG, Alfa Aesar, #A17925, Tewksbury, MA, USA) in 0.15 mol/L NaCl solution. An image of the water/air interface was taken at time zero, then at 1 min intervals for 5 min. Images were processed using a custom MATLAB script to determine fluid displacement rate for each measurement. This displacement rate was multiplied by the cross-sectional area of the tubing (internal diameter = 0.38 mm) to determine the fluid flux (*q*_*water*_) for all further calculations. Membrane permeability was determined from the following equation:

$$\frac{k_{mem}}{\mu_{.15\;\text{mol}/L\;\text{NaCl}}}=\frac{q_{\text{water}}\text{ln}\;\left(\frac{r_{2}}{r_{1}}\right)}{2\pi L\left(\pi_{PEG}\right)}.$$

For these permeability measurements, the pressure acting on the probe membrane was entirely due to the PEG-induced osmotic pressure of the equilibrium solution (*π*_*PEG*_), which was calculated from the virial coefficients measured at 25 °C (Chahine *et al*., 2005). The ion-driven osmotic pressure gradient was considered negligible because the concentration of NaCl on both sides of the membrane was equivalent. All solutions were allowed to come to room temperature prior to use, and each probe was labeled so that its respective permeability could be used to calculate swelling pressures from tissue flux measurements.

### Tissue flux measurements

NP tissue from the second caudal disc (C2/3) was dissected from 5 bovine tails (*N* = 5). The NP tissue from each IVD was then cut into 5 pieces (*n* = 25), weighed, and placed in dialysis tubing (1 kDa) with clips (Fig. 1). The 5 pieces of NP tissue from each disc (0.20 ± 0.07 g at excision) were distributed to 5, 10, 15, 20 and 25 % (g/mL) 20 kDa PEG in 0.15 mol/L NaCl solution. Osmotic pressures applied by means of PEG were calculated using virial coefficients as described above and ranged from 0.03 to 0.57 MPa. Tissue was loaded using equilibrium dialysis at room temperature with constant stirring for at least 60 h prior to flux measurements. Prior to taking flux measurements, micro-osmometer probes were prepared as described above. Dialysis bags containing the tissue pieces were then removed from the equilibration solution, patted dry, and clips were removed from the dialysis membrane, leaving the tissue loosely packed inside. Three flux measurements were taken from each piece of tissue, with the probe membrane placed in a different location each time. Images of fluid displacement were taken and processed as described above to determine flux. The average flux from each set of three measurements was used to calculate tissue swelling pressure. Finally, to measure the hydrostatic pressure within the tissue, a needle pressure transducer (Gaeltec, #CTN/4F-HP, Dunvegan, Scotland), was inserted into tissues spanning the range of applied osmotic pressures as described previously (McNally and Adams, 1992).

### Tissue hydration and mass loss ratio

After the three flux measurements, each tissue was placed on a scale to determine post-flux wet weight, and frozen at −80 °C. Tissues were then lyophilized for 48 h and their dry weights were measured. Tissue hydration at the end of equilibration was calculated on a total tissue water (TTW) basis, meaning that intra-tissue water located in both extrafibrillar and intrafibrillar compartments was included in the consideration of osmotic properties. Thus, the calculation for tissue hydration was

$$\frac{\text{(post flux weight }-\text{ dry weight) }}{\text{ dry weight }}.$$

### Intra-tissue sodium measurements

The ionic component of tissue swelling pressure, which arises from the concentration of sodium and chloride ions within the tissue, was determined independently from flux measurements. ICP-OES was used to measure intra-tissue sodium, while intra-tissue chloride concentrations were determined from Gibbs-Donnan theory, as described below. To measure intra-tissue sodium, lyophilized tissue (3 to 11 mg each) was weighed and then digested with 1 mL of ultrapure nitric acid in closed, acid cleaned DigiTubes (SCP Science, # 010-500-261, Quebec, Canada) using a DigiPREP MS graphite digestion block (SCP Science, # 010-500-205) (10 min at 95 °C). After cooling, the samples were diluted to 50 mL with deionized water. The sodium concentration in each sample was measured using a PerkinElmer Optima 4300DV (PerkinElmer, MA, USA) inductively-coupled plasma optical emission spectrometer, calibrated with sodium standard solutions containing 0, 0.5, 1, 2, 5 and 10 μg/mL sodium, made by dilution from a 1000 μg/mL sodium standard solution (Inorganic Ventures, #CGNA1, Christiansburg, VA, USA). The total mass of sodium measured per sample was converted to intra-tissue sodium concentration by normalizing to TTW volume.

Each tissue’s FCD and chloride content were then calculated using the partition coefficient (*k*), described by Gibbs-Donnan equilibrium, which represents the distribution of ions between the bath solution and tissue (Gooch and Tennant, 1997):

$$k=\frac{c_{-,ext}}{c_{-,int}}=\frac{c_{+,int}}{c_{+,ext}}.$$

In this equation, *c*_+,*int*_ & *c*_−,*int*_ denote concentrations of cations (Na^+^) and anions (Cl^−^) within the tissue, measured by ICP-OES and calculated using Gibbs-Donnan equations, respectively. *c*_+,*ext*_ & *c*_−,*ext*_ denote the concentration of the same ions in the equilibration solution, which were assumed unchanged by equilibration.

The requirement for charge neutrality in both the tissue and equilibration solution yields the tissue’s fixed charge density (*c*_*f*_),

$$c_{f}=kc_{+,ext}-\frac{c_{+,ext}}{k}.$$

Chloride concentration was computed as the difference between the intra-tissue sodium concentration measured from ICP-OES and the fixed charge density. Total ions were determined from the sum of this chloride concentration and sodium concentration measured from ICP-OES. Tissue ionic swelling pressures (*Δπ*_*t*,*ion*_) were then calculated using:

$$\Delta \pi_{t,ion}=\phi_{int}RT\left(c_{+,int}+c_{-,int}\right)-2\phi_{ext}RTc_{ext}=\phi_{int}RT\sqrt{c_{f}{}^{2}+4c_{ext}{}^{2}}-2\phi_{ext}RTc_{ext}.$$

Where *R* represents the universal gas constant, *T* represents temperature, and *∮* represents the osmotic coefficient, which describes the degree to which NaCl remains bound in solution. For this study, sodium ions were assumed to remain bound to negatively-charged proteoglycans with the same affinity they do to chloride ions, in accordance with previous studies (Maroudas and Evans, 1972; Gu *et al*., 1997). Therefore, the osmotic coefficient for NaCl (*∮* = 0.93) was applied to both the internal (tissue) and external (bath) ion concentrations.

Finally, tissue osmolalities were determined from the following equation, described by van’t Hoff (1887):

$$\phi_{int}\left(c_{+,int}+c_{-,int}\right)=\frac{\pi_{\text{tissue}}}{RT}.$$

Where (*π*_*tissue*_) represents the swelling pressure within the tissue, determined by flux or Gibbs Donnan theory.

### Statistics

All statistics were performed in Minitab, unless otherwise specified, with *α* = 0.05. The strength and direction of association between tissue hydration and applied pressure, as well as that between intra-tissue sodium ion concentration and applied pressure, were determined using Spearman’s correlations (Fig. 4a,b). Fits for the data presented in Figs 5b–c, were obtained using linear regression, and the *R*^*2*^ values presented describe the goodness-of-fit for each model. The fit for the data presented in Fig. 4c was obtained using nonlinear regression; however, because of its nonlinear nature, *R*^*2*^ could not be calculated for this model. Instead, the standard error of regression (*S*) was used, which denotes the average distance (in units of the dependent variable) between experimental data points and the calculated line, with a value of 0.0 indicating perfect fit. Applied pressures and measured swelling pressures were compared using Lin’s concordance correlation (Fig. 5a) in the statistical software R (Web ref. 1). The concordance correlation coefficient presented, ρ_*c*_, describes the strength of agreement between the compared variables, with perfect agreement indicated by a value of 1.0. The statistical significance of differences between the swelling pressures and osmolalities reported from flux and Gibbs-Donnan methods was determined from a two-way ANOVA, with Fisher *post hoc* analysis (Fig. 5b–d). Finally, the strength and direction of association between applied pressure and the percentage of the flux-based swelling pressures accounted for by Donnan swelling was determined from a Spearman correlation.

## Results

### Membrane, tissue, and total effective permeabilities

**Fig. 3.**
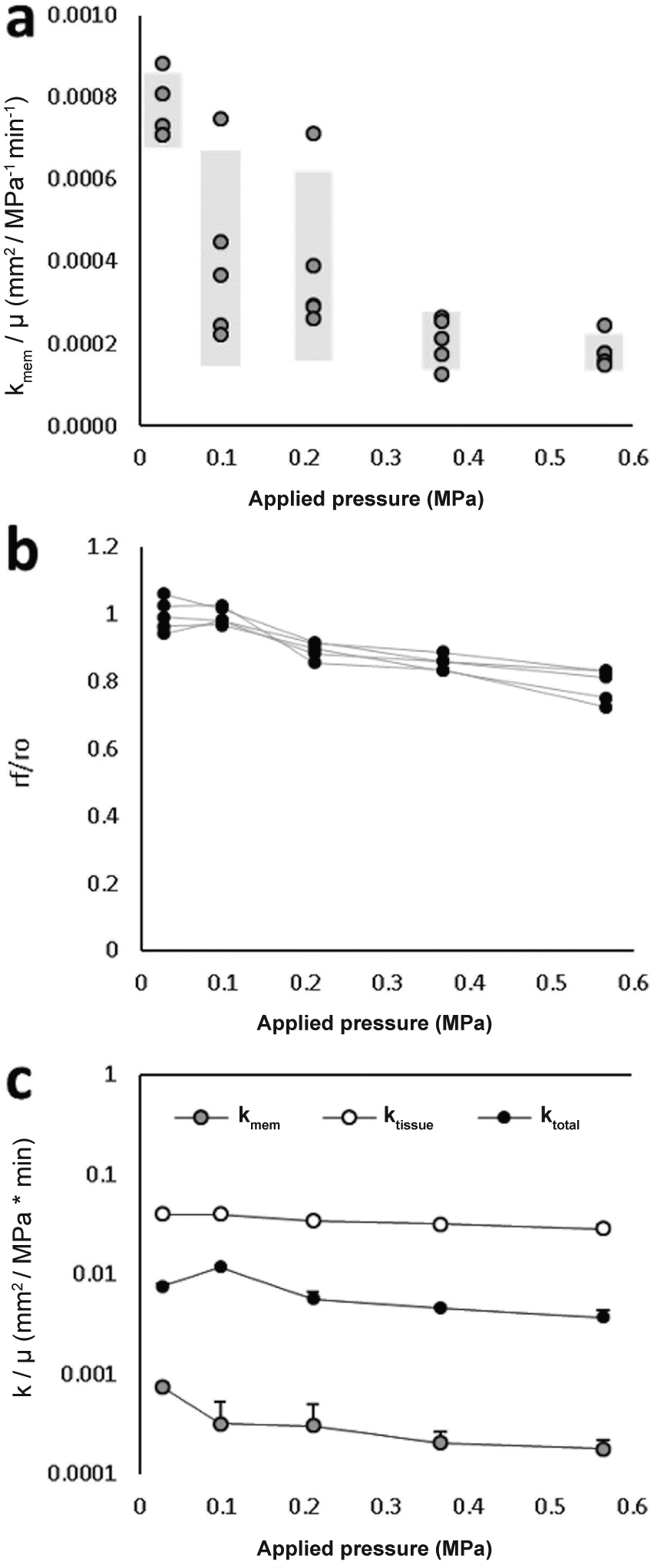
Membrane, tissue, and effective total permeabilities. (**a**) Probe membrane permeabilities measured under known osmotic pressures, applied using polyethylene glycol (PEG). Gray bars denote 95 % confidence intervals. (**b**) Radial stretch ratios used to approximate tissue permeability, given changes in porosity with applied pressure. (**c**) Membrane, tissue, and effective total permeabilities calculated at each applied pressure (averages with SD).

Probe membrane permeability generally decreased with applied pressure (Fig. 3a). However, there was significant variability between probes at the same applied pressure (Fig. 3a), which highlighted the necessity of matching observed fluxes with corresponding membrane properties.

At applied pressures less than 0.2 MPa, the radial stretch ratio was, on average, greater than 0.9, suggesting little water was expressed from the excised tissue (Fig. 3b). Beyond applied pressures of 0.2 MPa, tissue radius decreased with applied pressure in every case. Effective total permeability also generally decreased with applied pressure (Fig. 3c); however, there was an increase in effective total permeability between applied pressures of 0.03 and 0.09 MPa. This increase arose because a single probe with substantially higher permeability at each applied pressure was used for those measurements (probe corresponds to points outside the 95 % confidence intervals in Fig. 3a).

### Osmotic properties of the tissue

**Fig. 4.**
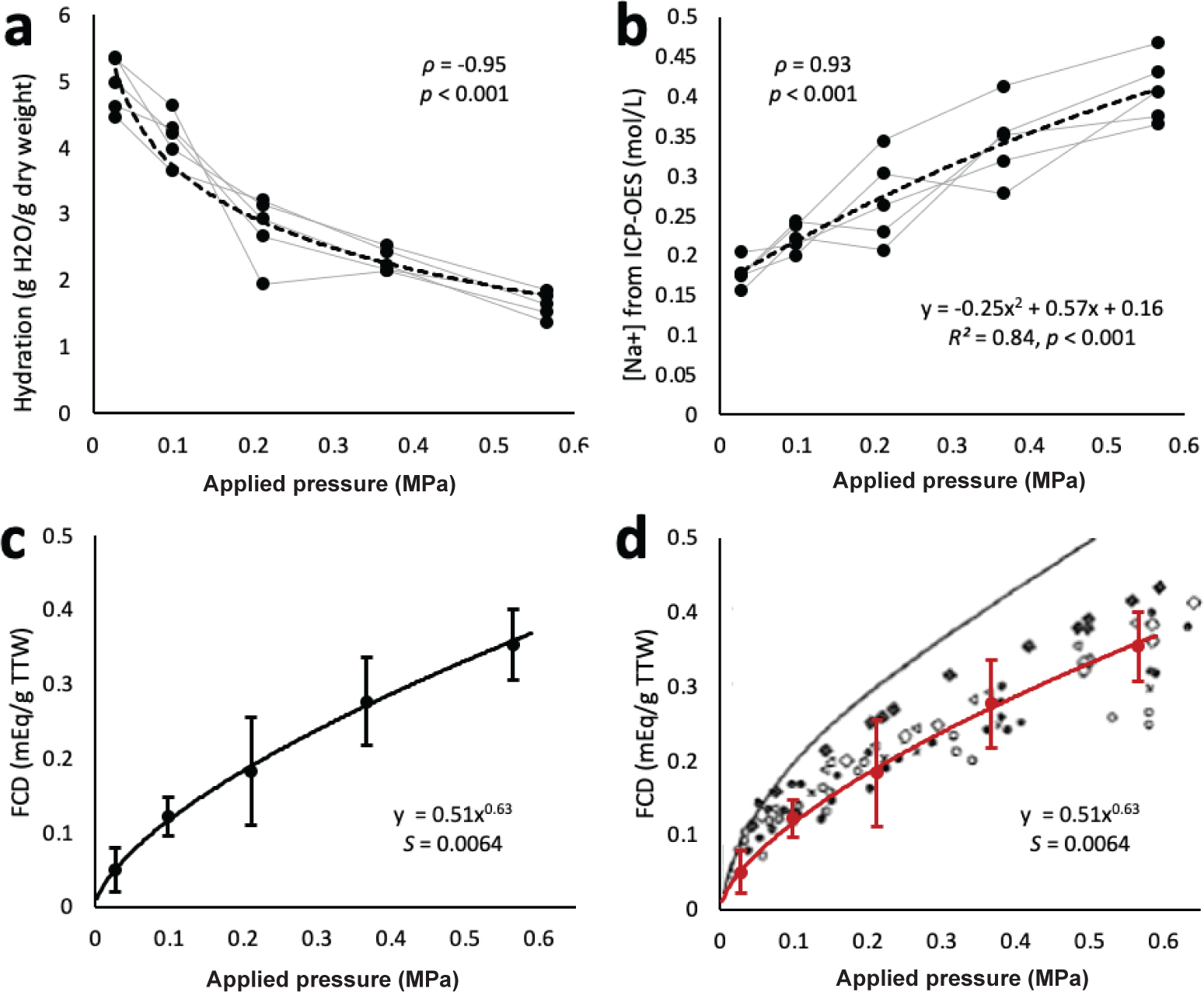
Tissue composition from ICP-OES and Gibbs-Donnan. (**a**) Tissue hydration following equilibration under osmotic pressure. (**b**) Intra-tissue sodium concentrations determined from ICP-OES and tissue hydration. (**c**) Tissue fixed charge densities for bovine NP in this study, calculated from Gibbs-Donnan equations. (**d**) Overlay of calculated fixed charge densities with those measured for human NP tissue. Black line represents FCD in mEq/EFW as presented by Urban and McMullin (1985). Figure reprinted from Urban JPG, McMullin JF (1985), with permission from IOS Press.

As expected, there was a significant negative correlation between tissue hydration and applied pressure (*ρ* = −0.95, *p* < 0.001, Fig. 4a), with tissue hydration decreasing as applied pressure increased. % H_2_O, calculated as 100 × *g H*_*2*_*O* / *g wet weight*, decreased linearly with applied pressure (*R*^*2*^ = 0.90, *p* < 0.001; data not shown). Correspondingly, the intra-tissue sodium ion concentration was positively correlated with applied pressure (*ρ* = 0.93, *p* < 0.001, Fig. 4b) and the relationship was described by:

$$\text{Intra-tissue }\left[{\text{Na}}^{+}\right](\text{mol}/L)=0.25\pi_{\text{PEG}}{}^{2}+0.57\pi_{\text{PEG}}+0.16\;\left(R^{2}=0.84,p<0.001\right).$$

The calculated tissue FCD increased with applied pressure according to a power law relationship, shown in Fig. 4c. This relationship in bovine NP tissue agreed well with that previously measured by Urban and McMullin (1985) in human tissue (Fig. 4d).

**Fig. 5.**
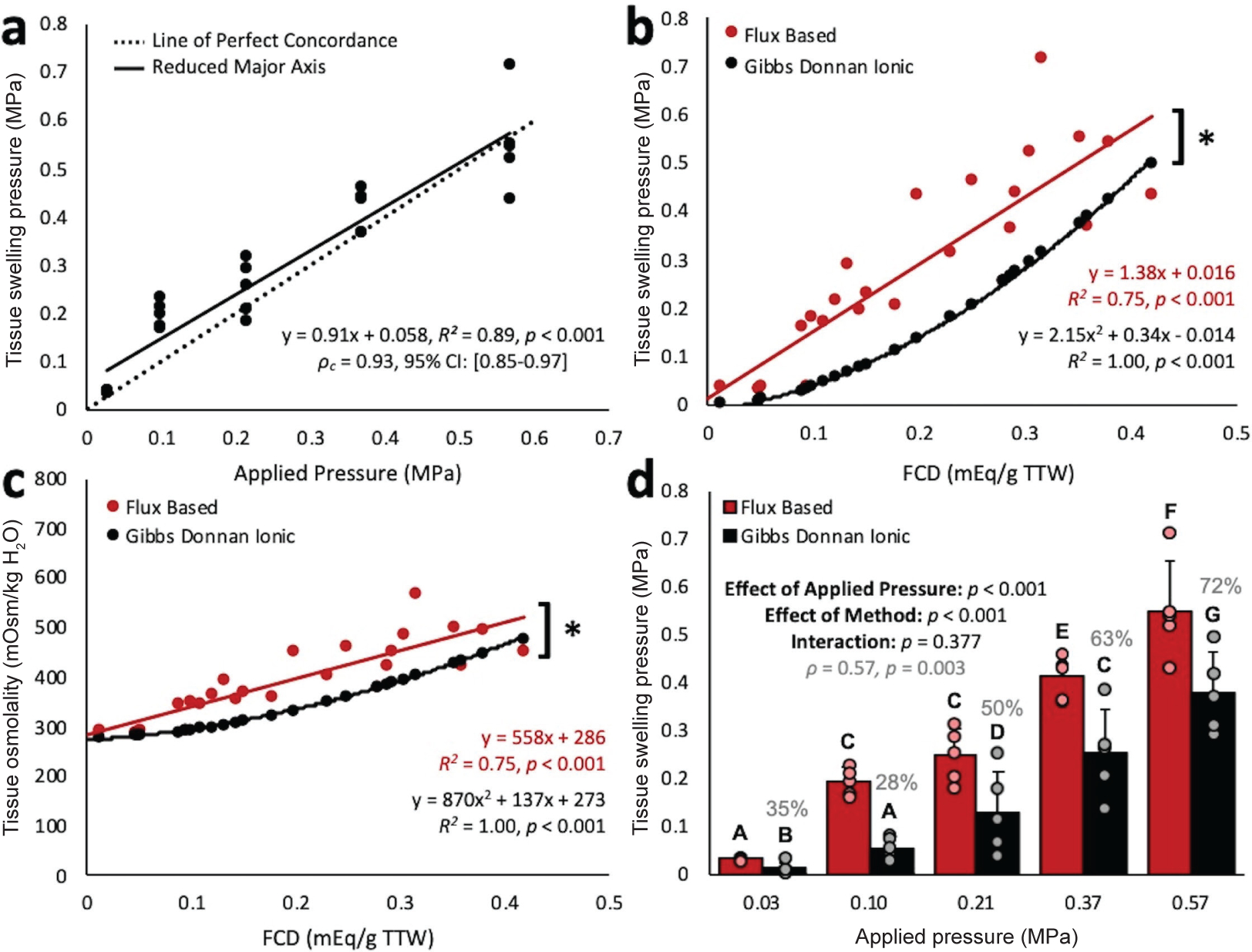
Tissue swelling pressures and osmolalities. (**a**) Concordance between total tissue swelling pressures calculated from flux measurements and osmotic pressures applied using PEG during equilibrium dialysis. (**b**) Comparison of swelling pressures from flux and Gibbs-Donnan based measurements, as a function of FCD. (**c**) Concentration of tissue osmolytes calculated from flux measurements and Gibbs-Donnan equations. (*) indicates significant effect of method for Two-Way ANOVA, *p* < 0.001 (flux-based *vs.* Gibbs-Donnan ionic). (**d**) Comparison of swelling pressures from each method. Groups that do not share a letter are significantly different. Percentages indicate the portion of the flux swelling pressures accounted for by Donnan swelling.

Tissue swelling pressures calculated from flux (Fig. 5a) increased linearly with the osmotic stress applied to the tissue during equilibration (*R*^*2*^ = 0.89, *p* < 0.001). The Lin’s concordance correlation coefficient for these pressures was 0.93, indicating moderate agreement. Additional consideration was given to the flux measurements in tissues equilibrated under 0.1 MPa because one probe, which had a permeability much greater than all other probes, was used for these measurements. Tissue swelling pressures seemed to be strongly influenced by this elevated permeability, possibly due to a small tear or other mechanical dysfunction within the probe membrane. With tissues equilibrated at 0.1 MPa excluded, the Lin’s concordance correlation coefficient increased to 0.95, indicating substantial agreement between applied pressures and calculated swelling pressures.

There was a significant linear relationship between flux-based tissue swelling pressure and FCD (*R*^*2*^ = 0.75, *p* < 0.001, Fig. 5b). The calculated Gibbs-Donnan ionic swelling pressure also increased with FCD. However, there were significant differences between flux-based and ICP-OES derived swelling pressures (Fig. 5b, *p* < 0.001) and osmolalities (Fig. 5c, *p* < 0.001). Here, osmolalities calculated from flux measurements increased linearly with FCD (*c*_*f*_) as described by:

$$\text{Tissue Osmolality }\left(\frac{\text{mOsm}}{kg\;H_{2}O}\right)=588c_{f}+286\;\left(R^{2}=0.75,p<0.001\right).$$

There was a significant effect of applied pressure on the measurements of intra-tissue swelling pressure and osmolality from both methods (Fig. 5d, *p* < 0.001). The interaction between applied pressure and method was significant for neither swelling pressures (*p* = 0.377) nor osmolalities (*p* = 0.051). From applied pressures of 0.21 to 0.57 MPa, the magnitude of change in tissue osmolality was 122 mOsm/kg H_2_O (Table 1). Extrapolating the data for osmolality to the range of pressures commonly used to simulate the diurnal cycle (0.2 MPa – 0.6 MPa) (Wilke *et al*., 1999), tissue osmolalities were 376 and 522 mOsm/kg H_2_O, corresponding to a magnitude of 146 mOsm/kg H_2_O.

The mass of water perfused during flux measurements accounted for 0.23 ± 0.14 % of the mass of TTW, and was therefore assumed to negligibly alter tissue hydration and osmotic pressure. Finally, the % change in Na^+^ and Cl^−^ concentration from the start of equilibration to the end was 0.39 ± 0.06 % and 0.12 ± 0.08 %, respectively, so the assumption required for Gibbs-Donnan equations (0.15 mol/L bath concentration at equilibrium) was considered reasonable.

## Discussion

The first aim of this study was to determine whether Darcy’s law for radial flow could describe the relationship between fluid flux and the swelling pressure of isolated NP tissue. Results demonstrated a significant linear correlation (*R*^*2*^ = 0.89, *p* < 0.001) between applied pressures and tissue swelling pressures, and suggested that these pressures balanced each other at equilibrium (ϱ_*c*_ = 0.93, Fig. 5a). These findings are consistent with previous studies in disc and cartilage, which have demonstrated that swelling pressure accounts for 95 – 100 % of the applied stress at equilibrium in healthy tissue (Urban and Maroudas, 1980; Urban and McMullin, 1985; Johannessen and Elliott, 2005; Canal Guterl *et al*., 2010; Quiroga *et al*., 2017). Therefore, results suggest this minimally invasive technique can be used to approximate tissue swelling pressures and osmolalities *in situ*. Furthermore, because the total pressure acting on the membrane drives flux, this technique, in combination with a needle pressure transducer, has the potential to differentiate hydrostatic and osmotic contributions to total fluid pressure within the IVD (Park *et al*., 2003). Altogether, this validation provides the groundwork for future use of the method to better elucidate the mechanisms of load sharing within the disc (*i.e*., hydrostatic pressure, osmotic pressure, and solid matrix stress). Importantly, this technique could be applied to whole-disc motion segments, and would enable measurements during transient loading periods, within both annulus fibrosus and nucleus pulposus regions as well as across stages of degeneration in human tissue.

To the best of our knowledge, the results reported here are the first direct measurements of intra-tissue osmolality for IVD tissue (Fig. 5c, Table 1). The tissue osmolalities that developed from pressures commonly used to simulate the diurnal cycle, 0.2 MPa – 0.6 MPa, (Wilke *et al*., 1999; Jünger *et al*., 2009; Paul *et al*., 2012; Walter *et al*., 2014) were 376 and 522 mOsm/kg H_2_O, respectively. This corresponded to a magnitude of 146 mOsm/kg H_2_O, which closely mirrored previous estimates for the magnitude of the diurnal cycle (400 – 550 mOsm/kg H_2_O), obtained from approximations of daily fluid loss (Sivan *et al*., 2006b). However, it is important to emphasize that the magnitude reported here represents tissue at equilibrium with the applied load. Multiple groups have demonstrated that the permeability of the disc prevents the tissue from reaching equilibrium within the time-course of a typical day (Vergroesen *et al*., 2016; Urban and McMullin, 1985). Therefore, in this study, the osmolalities measured for relatively healthy tissue at equilibrium likely overestimate the magnitude of the osmotic cycle that would develop from a typical diurnal loading schema (8 h, 0.2 MPa; 16 h, 0.6 MPa). Despite this limitation, the equilibrium-bounded magnitude (146 mOsm/kg H_2_O) provides an initial estimate for the diurnal cycle, and may help inform studies of osmotic mechanotransduction.

Both the magnitude and time-course of the disc’s diurnal osmotic cycle may vary considerably across the population and lifespan in humans. This heterogeneity has not yet been captured using existing measurement methods, and therefore has not yet translated to studies of cellular osmotic mechanotransduction. The absence of such measurements poses significant barriers to the identification of osmotically-driven cellular changes involved in the initiation and progression of disease, as well as to the associated development of therapeutic interventions. While there are currently obstacles to measuring diurnal swelling *in vivo*, improvements to experimental models may enable *in situ* measurements to better capture *in vivo* conditions. The osmotic cycle is regulated by both the extent of tissue degeneration - which varies based on age, injury, and skeletal maturity - and applied loads - which vary based on body mass index (BMI) (Samartzis *et al*., 2012), physical activity (Bowden *et al*., 2018), and muscular tension (Granata and Marras, 1993; Imamura *et al*., 2017). Therefore, *in situ* experiments that pair donor tissue with load magnitudes, informed by these donor-specific parameters, may provide truer measures for the magnitude and time-course of osmotic changes that the disc and its cells experience *in vivo*. Ultimately, the micro-osmometer technique described here has the potential to make such measurements, which would help inform the design of cellular studies to determine downstream effects of tissue loading on a more clinically relevant basis.

The second aim of this study was to evaluate Gibbs-Donnan equations directly for their capability to approximate osmotic swelling within the NP. The ICP-OES results from the present study demonstrated that Donnan swelling pressures based on TTW were significantly different from swelling pressures measured using flux (Fig. 5b, *p* < 0.001), accounting for as little as 35 % of the flux based swelling pressure at low applied loads (0.03 MPa) and 72 % of the total at high applied loads (0.57 MPa). Assuming that micro-osmometer flux is driven by the total swelling pressure within the tissue, these results are consistent with previous studies demonstrating that Donnan estimates do not alone capture the tissue’s propensity to swell, due to the existence of non-ionic osmotic pressures (Urban *et al*., 1979a; Lai *et al*., 1991; Kovach, 1995). Here though, the underestimation of the total swelling pressure by Gibbs-Donnan is also influenced by the normalization of FCD to TTW. Consistent with many benchtop and modeling studies, the osmotic properties of the tissue were normalized (*e.g*., FCD, ion content, and osmotic pressure) to TTW (Perie *et al*., 2006; Iatridis *et al*., 2007; Massey *et al*., 2012; Ko and Quinn, 2013; Gu *et al*., 2014; Zhu *et al*., 2014; Wu *et al*., 2017). However, because the disc’s fibrillar collagens bind water, only a fraction of the tissue’s total water - the extrafibrillar water (EFW) - is available for osmotic exchange (Urban and McMullin, 1985; Urban and McMullin, 1988). Therefore, normalizing the obtained ICP-OES results instead to this EFW would increase Gibbs-Donnan swelling pressures and osmolalities toward those measured from flux. These results highlight that normalization to TTW may cause significant underestimation of the tissue’s Donnan swelling pressure, which is widely considered the major source of swelling within the NP.

Many finite element models of the IVD utilize FCD normalized to TTW, and have demonstrated that incorporating osmotic pressures using this approach provides an improved framework to model whole disc mechanics (Wilson *et al*., 2005). However, our results support previous findings that normalizing to TTW, rather than EFW, alters the apparent balance of load-bearing mechanisms within the disc, reducing osmotic pressures and increasing matrix stresses within the NP (Schroeder *et al*., 2007). Results further demonstrated that the difference between Gibbs-Donnan swelling pressures (based on TTW) and those measured using flux decreased with increasing load (*p* = 0.003). Studies of PG solutions have previously demonstrated that the non-ionic component of swelling pressure increases with load (Urban *et al*., 1979a; Kovach, 1995; Chahine *et al*., 2005). Therefore, in tissue - when osmotic properties are normalized to EFW - non-ionic swelling would be expected to cause an increase - rather than a decrease - in the discrepancy between ionic and total swelling pressures with compression. Instead, the convergence between Donnan and total swelling pressures observed in this study may result from compression-induced changes in EFW volume. This interpretation is consistent with previous studies which demonstrated that compression reduces the d-spacing within collagen fibrils, causing water to be expelled from the intrafibrillar space (Sivan *et al*., 2006a). Therefore, with greater tissue compaction, more of the tissue’s water is extrafibrillar, and the TTW-based Donnan swelling measurement is expected to be closer to its true, EFW-based value. Altogether, results highlight that incorporating the exchange of water between intrafibrillar and extrafibrillar space is necessary to accurately assess the physiologic osmotic environment. Accordingly, benchtop and modeling studies that utilize TTW-based measures of FCD may miscalculate the evolution of individual load-sharing mechanisms and the biological environment experienced by embedded cells. Therefore, these differences in osmotically active water are important considerations for experiments which aim to measure or model osmotic properties.

Aside from EFW considerations, differences between swelling pressures measured using flux and ICP-OES emphasize the importance of the non-ionic contribution to the total osmotic pressure within the IVD. Studies have suggested that in the NP non-ionic sources of osmotic pressure balance 12–15 % of the applied stress (Urban *et al*., 1979a; Heneghan and Riches, 2008b). It is generally assumed that these non-ionic osmotic pressures result primarily from the tissue’s PGs, with contribution from collagens being considered negligible (Kovach, 1995). Existing mathematical descriptions for the non-ionic component, therefore, reflect PGs in solution and have been confirmed for such solutions by experimental data (Urban *et al*., 1979a). However, they have not yet been confirmed for tissue. Currently, in tissue, the method used to separate ionic and non-ionic swelling involves sequential compression testing in hypertonic and isotonic baths (Lu *et al*., 2004; Heneghan and Riches, 2008b, Flahiff *et al*., 2004). In this method, the operating understanding is that sufficiently hypertonic conditions engender large intra-tissue ion concentrations, which overwhelm the ion concentration gradient that develops in a physiological environment due to FCD and electroneutrality requirements. Therefore, mechanical properties measured in a hypertonic bath are assumed to result purely from non-ionic effects. However, studies have suggested that the large intra-tissue ion concentrations required for this method alter configurational entropy within the tissue, due to changes in charge shielding and PG self-repulsion (Chahine *et al*., 2005). Because configurational entropy is generally considered the largest source for non-ionic osmotic pressure (Kovach, 1995), this method may not provide a reliable measurement for non-ionic swelling. Combined, the two techniques presented in this study - micro-osmometer flux and ICP-OES (with normalization to EFW) - provide an alternative method to measure non-ionic swelling pressures within tissue, and may help develop a more complete understanding of osmotic behavior in cartilage and the IVD.

In addition to the limitations previously discussed, it’s important to note that the tissue permeability equation used in the application of Darcy’s law was validated in bovine NP tissue under confined compression (Heneghan and Riches 2008a), while this study applied pressures isotropically by equilibrium dialysis. Extrapolation of this equation from 1D stretch to 3D stretch, combined with the assumptions required to obtain a radial stretch ratio, likely influenced the results. Furthermore, the perfused volume of tissue was assumed to be cylindrical and equivalent to the volume of fluid perfused. This simplification does not account for the space occupied by other species within the tissue. However, given that the timescale of the measurement (5 min) was much smaller than the time required to reach internal equilibrium, the perfused fluid had comparably negligible time to redistribute within the tissue, and the assumption was considered reasonable. Similarity between equilibrium swelling pressures presented here and those previously published (Urban and Maroudas, 1980; Urban and McMullin, 1985; Johannessen and Elliott, 2005; Sivan *et al*., 2006a; Canal Guterl *et al*., 2010; Quiroga *et al*., 2017) suggests that these assumptions required to derive intra-tissue swelling pressures were reasonable. Additional consideration was given to the use of bovine tail discs, which may incur different loads than human tissue *in vivo*. (Reitmaier *et al*., 2017; Fusellier *et al*., 2020). However, previous studies have suggested that caudal bovine NP tissue provides a reasonable model for biochemical properties and *in situ* mechanical testing, as its PG and water contents are comparable to that in human lumbar NP tissue (Oshima *et al*., 1993; Demers *et al*., 2004; van Dijk *et al*., 2011). In agreement with these studies, the FCD measurements of bovine NP tissue presented here correlated well with those from human tissue (Urban and McMullin, 1985). Combined, these results suggest that the equilibrium swelling pressures and equilibrium-bounded intra-tissue osmolalities measured in this study provide a reasonable estimate for corresponding equilibrium values in excised human NP tissue.

Overall, this study demonstrated that tissue swelling pressures could be measured using the micro-osmometer and system of equations described herein. Results also affirm previous suggestions that constitutive models, through normalization to TTW and incorporation of only the ionic component of swelling pressure, may currently be underestimating the osmotic contribution to the IVD’s mechanical behavior. Future applications of the micro-osmometer and ICP-OES techniques will enable a more direct characterization of the non-ionic contribution to tissue swelling. The micro-osmometer technique itself has the potential to be applied *in situ* for whole-disc motion segments under dynamic conditions, and therefore could provide a greater understanding of the diurnal osmotic cycle (the range of magnitudes and rates experienced) as it changes with disease. Ultimately, such measurements could help establish a more comprehensive paradigm for studies of cellular mechanotransduction in health and disease.

## Figures and Tables

**Table 1.** Changes in the osmotic properties of bovine NP tissue with applied pressure.

Applied pressure (MPa)	Hydration (g H_2_O/g dry weight)	From ICP-OES, based on TTW				From flux	
		[Na^+^] (mol/L)	[Total ions] (mol/L)	FCD (mEq/g TTW)	Tissue osmolality (mOsm/kg H_2_O)	Tissue osmolality (mOsm/kg H_2_O)	Δ osmolality from 0.21 mpa
**0.03**	4.96 ± 0.41	0.18 ± 0.02	0.31 ± 0.01	0.05 ± 0.03	284 ± 5^B^	293 ± 2^AB^	−87 ± 23
**0.10**	4.16 ± 0.37	0.22 ± 0.02	0.32 ± 0.01	0.12 ± 0.03	302 ± 9^A^	357 ± 11^C^	−22 ± 26
**0.21**	2.78 ± 0.51	0.27 ± 0.06	0.36 ± 0.04	0.18 ± 0.07	331± 35^D^	379 ± 22^C^	0
**0.37**	2.30 ± 0.17	0.34 ± 0.05	0.41 ± 0.04	0.28 ± 0.06	381 ± 37^C^	445 ± 18^EF^	66 ± 35
**0.57**	1.64 ± 0.37	0.41 ± 0.04	0.46 ± 0.04	0.35 ± 0.05	432 ± 34^F^	502 ± 41^E^	122 ± 40

* For those values marked with superscripted letters: groups that do not share a letter were identified as significantly different by Two-Way ANOVA with Fisher *post-hoc* comparisons.

## Discussion with Reviewers

Theo Smit: In the experiments, stress is applied by submerging the NP samples in high-osmotic media. Furthermore, the NP samples are unconfined. How does this compare to the NP *in situ* (or *in vivo*), which is confined by the AF and end plates and subjected to spinal compression?

Authors: This study was designed as a proof-of-concept for the method. Assuming that the tissue had come to equilibrium with the applied loads, even if the loads were applied under confined compression, we do not think that the flux measurements would yield significantly different results. This is because the FCD, which is the driver of osmotic pressure and therefore flux, is dependent primarily on the degree of tissue compaction. Therefore, we expect that the pressures applied osmotically would yield tissue osmolalities comparable to those that would be produced under equivalent mechanical pressures.

Theo Smit: How would the osmolality of the AF affect the equations and outcomes of the study?

Authors: The inclusion of the AF would not affect the equations used to derive measures for the osmotic environment. The primary factors that would influence the outcomes of the described method would be the pressure gradients present across the membrane, meaning the conditions in the tissue immediately surrounding where the probe is placed. This method could be applied to other tissues (including the AF). The osmolality of the AF itself would not affect the measurements in this study - importantly which were performed at equilibrium - as both NP and AF regions would necessarily contain the same osmotic environment.

Theo Smit: Why was it assumed that ions do not cross the membrane which has a barrier only for 6 kDa molecules? Same question for the tissue, which is even more porous.

Authors: There is certain to be some diffusion of ions between the probe and tissue. However, we expect the net diffusion of ions to be nearly zero. The tissue, since it is assumed to be at equilibrium, is understood to be electrically neutral. Since it was equilibrated in a 0.15 mol/L NaCl solution, the net diffusion of ions with a new 0.15 mol/L NaCl solution is expected to be nearly zero as it was already in equilibrium with a 0.15 mol/L NaCl solution. The reviewer raises an important distinction, however, between this proof-of-concept study performed at equilibrium and future studies which may be performed under transient conditions. Under transient conditions, one would have to assume that the flux of ions during the time scale of the measurement (5 min) would be negligible in reference to the total time remaining until equilibrium.

Theo Smit: What is the role of tissue stiffness and elasticity in this model? Withdrawing water from the tissue induces elastic energy, which in turn could drive fluid flow when stress is released.

Authors: We believe the method used here can be described by the relationship between the fluid pressures within the tissue and fluid flux, according to Darcy’s law. We expect that the stresses in the solid matrix, to which the reviewers refer, would only influence the results of our study in so far as they alter the fluid pressures acting on the membrane. In the situation when an applied stress is released or reduced, we expect fluid flux through the microdialysis probe to be driven by changes in intra-tissue hydrostatic pressure and osmotic pressure, which can be measured as described.
